# Emerging tobacco use patterns in the United States: Tobacco use behaviors associated with current use of nicotine pouches

**DOI:** 10.1371/journal.pone.0343111

**Published:** 2026-03-02

**Authors:** Julia N. Soulakova, Lisa J. Crockett

**Affiliations:** 1 Department of Population Health Sciences, College of Medicine, University of Central Florida, Orlando, Florida, United States of America; 2 Department of Psychology, University of Nebraska-Lincoln, Lincoln, Nebraska, United States of America; University of Saskatchewan, CANADA

## Abstract

Nicotine pouches (NPs) are non-combustible tobacco products marketed as alternatives to traditional tobacco. Recent studies suggest an increasing use of NPs in the United States. The primary goals of this study were to assess the associations between current use of each of the seven other tobacco products and current use of NPs among U.S. adults (18+), and to determine which other products are significantly associated with current use of NPs. The secondary goals were to describe the population of adults who currently use NPs in terms of their tobacco use behaviors and sociodemographic characteristics. Data from the 2022–2023 Tobacco Use Supplement (TUS) to the Current Population Survey (CPS), collected in the U.S., were used in this cross-sectional study to address the primary (n = 107,281) and secondary (n = 537) goals. The primary outcome was current NP use (yes/no). All analyses accounted for the TUS-CPS complex design. The prevalence of current NP use was 0.47% (SE = 0.03%), corresponding to an estimated 1,146,681 adults nationwide. Adults who currently used smokeless tobacco (aOR=12.03, 95% CI = 8.83-16.39) had the highest odds of current use of NPs, followed by those who used heated tobacco (aOR=4.84, 95% CI = 2.01-11.67), hookah tobacco (aOR=3.70, 95%CI = 1.55-8.82), e-cigarettes (aOR=2.90, 95% CI = 1.84-4.60), cigars (aOR=2.48, 95% CI = 1.71-3.58), and combustible cigarettes (aOR=1.84, 95% CI = 1.38-2.45). Among adults who currently use NPs, 61.00% (SE = 2.59%) used at least one other tobacco product and the most commonly used products were smokeless tobacco (27.39%), combustible cigarettes (21.74%), e-cigarettes (20.54%), and cigars (17.30%). The observed sociodemographic profile of adults who use NPs (e.g., 89.14% are men) suggests that NPs may be reaching subgroups that differ from those who typically use other tobacco products. The findings underscore the need for further research to determine whether use of NPs leads to product switching, cessation, or sustained poly-tobacco use.

## Introduction

Nicotine pouches are a new class of non-combustible tobacco products marketed as alternatives to traditional tobacco [1]. Although they are used orally in a manner similar to traditional smokeless tobacco products (such as snus), nicotine pouches do not contain tobacco leaf. In the U.S., they are regulated as tobacco products because they contain nicotine (derived from tobacco or synthetically). Nicotine pouches were first introduced to the U.S. market in 2014 with the launch of the Zyn brand, followed by the introduction of two additional brands in 2016, and several more in subsequent years [2].

Although little is known about the health impact and long-term effects of nicotine pouch use, nicotine pouches are perceived by some to pose lower health risks than cigarettes because they do not contain tobacco leaf or its associated carcinogens [1–3]. Nonetheless, concerns have been raised regarding their potential to cause nicotine addiction and adverse effects on cardiovascular and oral health [1,3]. Furthermore, the rising popularity of nicotine pouches [4], has generated additional public health concerns.

A few recent population-based studies have examined nicotine pouch use among U.S. adults. For example, in 2022–2023, the prevalence of current use was 0.4% [5,6], while the prevalence of past 30-day use was 0.8% [7]. Current use of nicotine pouches was more common among adults who currently smoked cigarettes or used e-cigarettes, or who had ever used smokeless tobacco, compared with adults who had never used these products [5,6]. In addition, the past 30-day use was more common among adults who currently used smokeless tobacco (11.8%), e-cigarettes (4.2%), or combustible cigarettes (1.5%) relative to those who had never used the respective product [7]. Nonetheless, use of other tobacco products, such as cigars, heated tobacco, hookah tobacco, and regular tobacco pipes, has not been examined in the context of nicotine pouch use.

Prior studies have also identified significant differences in nicotine pouch use by sociodemographic characteristics. For example, the prevalence of current pouch use was higher among younger adults (18–24, 25–44, and 45–64 years) compared with adults aged 65 and older, among men relative to women, and among non-Hispanic White adults compared with communities of color [5,6]. In addition, sociodemographic characteristics have been described for adults who use other emerging tobacco products such as e-cigarettes and hookah tobacco [8]. However, the sociodemographic characteristics and tobacco use behaviors of adults who currently use nicotine pouches remain largely unknown.

The **primary goals** of this study were to assess the associations between current use of other tobacco products (including cigars, combustible cigarettes, e-cigarettes, heated tobacco, hookah tobacco, regular tobacco pipes, and smokeless tobacco) and current use of nicotine pouches among U.S. adults (18 + years old), and to identify which products are significantly associated with nicotine pouch use. When addressing these goals, we considered several factors that may influence tobacco use [9–11], including nicotine pouch use [5,7], among adults: (1) sociodemographic characteristics including age, biological sex, race/ethnicity, marital status, highest level of education, annual family income, employment status, metropolitan area of residence, and U.S. region, and (2) the survey mode (phone, in-person) [12,13]. Based on findings from two prior population-based studies [5,7], we **hypothesized** that:

### Hypothesis 1:

For each tobacco product considered, the prevalence of current use of nicotine pouches will be significantly higher among adults who use the product compared to those who do not, and

### Hypothesis 2:

Among the tobacco products considered, current use of smokeless tobacco will be associated with the highest prevalence of current use of nicotine pouches.

The **secondary goals** were to describe the population of adults who currently use nicotine pouches in terms of their other tobacco use behaviors and sociodemographic characteristics. We also aimed to determine whether tobacco use behaviors of adults who use nicotine pouches differ from those observed in the overall adult population, and whether their sociodemographic characteristics differ from those observed for adults who use other emerging tobacco products, such as e-cigarettes or hookah tobacco [8].

## Materials and methods

### Data source and study measures

We merged data from three monthly waves of the 2022–2023 Tobacco Use Supplement to the Current Population Survey (TUS-CPS): September 2022, January 2023, and May 2023 [14,15]. The Supplement is administered every four years and provides key information on tobacco use behaviors in the U.S. adult population (aged 18 years and older). We accessed the publicly available data on March 4, 2025. The study dataset included responses from 107,281 adults with complete information on all sociodemographic and tobacco use measures considered in the study. [The initial TUS-CPS sample included 107,455 adults with complete information on current use of nicotine pouches, the primary study outcome. Among those, data were missing for 174 (0.16%) adults for at least one independent variable, resulting in a final analytic sample of 107,281 adults.]

The **key dependent variable** was current nicotine pouch use, recorded as a binary indicator (yes/no). This measure was defined by combining responses to two survey questions, regarding ever use (yes/no) for all respondents, and current use (daily, someday, not at all) for those who reported ever using nicotine pouches. As a result, the current nicotine pouch use measure differentiated between adults who reported daily or occasional use and those who reported never or former use. We note that the TUS-CPS questionnaire distinguishes nicotine pouches from smokeless tobacco products by clarifying that pouches do not contain tobacco leaf.

The **independent variables** included seven indicators of current use of other tobacco products, assessed separately for: (1) cigars (such as regular cigars, cigarillos, or little filtered cigars), (2) combustible (regular) cigarettes; (3) e-cigarettes (such as vape-pens, e-hookahs, vapes, or modes); (4) heated tobacco (aka “heat not burn” tobacco products that use capsules or sticks to produce vapor, rather than e-liquids used in e-cigarettes), (5) hookah tobacco (such as water or hookah pipes filled with tobacco), (6) regular pipes filled with tobacco; and (7) smokeless tobacco (such as moist snuff, dip, spit, or chew tobacco, or snus). Current use of each of the seven tobacco products was defined as “current use” or “non-use” similar to the measure for current nicotine pouch use, with one exception: for combustible cigarettes, the initial survey question assessed lifetime smoking of 100 or more cigarettes (rather than any use). In addition, we created a binary variable “current use of at least one tobacco product” to distinguish adults who reported current use of any of these seven products from those who reported no current use. Other **independent variables** included sociodemographic characteristics and survey mode, as detailed in Table 1.

**Table 1:** Description of the overall sample (n = 107,281; N = 241,807,760). Analysis of 2022−23 Tobacco Use Supplement to the Current Population Survey data.

Characteristic	Sample Count	Weighted Percent^a^ (%)
**Age**		
18–24	5,462	11.93
25–44	33,531	34.25
45–64	34,269	31.78
65+	34,019	22.04
**Sex**		
Female	57,566	51.13
Male	49,715	48.87
**Race/Ethnicity** ^**b**^		
Hispanic	12,066	17.08
Non-Hispanic (NH) American Indian/Alaska Native	916	0.73
NH Asian/Asian American	5,095	6.31
NH Black/African American	10,180	12.03
NH Hawaiian/Pacific Islander	335	0.31
NH Multiracial	1,415	1.64
NH White	77,274	61.89
**Marital Status**		
Married (living with a spouse)	55,446	49.99
Widowed, divorced or separated	27,077	19.78
Never married	24,758	30.23
**Highest Level of Education**		
Less than high school	7,445	8.72
High school or equivalent	27,728	28.80
Some college or bachelor’s degree	55,311	49.15
Graduate degree or equivalent	16,797	13.32
**Employment Status**		
Employed	62,474	62.89
Unemployed	2,056	2.42
Not in labor force	42,751	34.69
**Annual Family Income**		
Less than $20,000	11,968	9.73
$20,000 – $39,999	19,343	16.36
$40,000 – $74,999	28,053	24.83
$75,000+	47,917	49.08
**U.S. Region of Residence**		
Northeast	17,045	17.38
Midwest	23,104	20.91
South	38,767	38.39
West	28,365	23.32
**Metropolitan Area of Residence**		
Metropolitan area	85,095	86.43
Non-metropolitan area	21,114	12.76
Unknown	1,072	0.81
Survey Mode^c^		
In-person interview	67,062	62.09
Phone interview	39,888	37.91

^a^ Percent is based on the corresponding population count; the total population count N = 241,807,760.

^b^ Due to insufficient sample sizes, NH American Indian/Alaska Native, NH Asian/Asian American, NH Hawaiian/Pacific Islander, and NH Multiracial groups were combined into a single “NH Other” category for the subsequent analyses.

^c^ Survey mode is available for 106,950 respondents only.

### Statistical analyses

To address the **primary goals**, we used the entire sample to perform (1) bivariate analyses comparing the prevalence of current nicotine pouch use between adults who use a given tobacco product and those who do not, testing for significant differences; and (2) model-assisted (i.e., multivariable) analyses estimating the odds ratios of current use of nicotine pouches among adults who use each tobacco product compared to those who do not, adjusting for sociodemographic characteristics and current use of the other tobacco products. Bivariate analyses were conducted using Rao-Scott chi–squared tests at 5% significance level [16]. The model-assisted analysis involved a design-specific multiple logistic regression model that was used to estimate the odds of current nicotine pouch use, where all independent variables (except survey mode) were included as main effects. The significance level was 5%. In addition, 95% simultaneous confidence bands were computed for significant predictors with multiple categories using Bonferroni adjustments. For example, for a predictor with four categories, three individual 98.33% confidence intervals were computed to compare each non-reference group to the reference group.

To assess the **secondary goals**, we restricted the sample to adults who currently used nicotine pouches (n = 531). This reduced sample was used to describe the subpopulation in terms of current use of other tobacco products and sociodemographic characteristics. Tobacco use patterns in this subpopulation were compared with those observed in the overall adult population (n = 107,281), while sociodemographic characteristics were compared with those of adults who use other emerging tobacco products, as reported in the literature [8]. We note that these analyses should be interpreted as descriptive.

All analyses included TUS-CPS survey weights and Balanced Repeated Replications (with Fay coefficient of 0.5) for variance estimation [17]. Analyses were conducted using the SAS®9.4 package [18]. The study dataset and SAS code can be found in Harvard Dataverse [19].

## Results

### Primary goals: current use of other tobacco products as correlates of current use of nicotine pouches

The study sample (n = 107,281) was representative of the 241,807,760 adults in the U.S. population. A description of the sample is provided in Table 1. The prevalence of current use of nicotine pouches was 0.47% (SE = 0.03%).

Table 2 presents the results of the bivariate analyses. For all seven tobacco products, current use of the product was significantly positively associated with current nicotine pouch use (all p-values<0.001). These findings support **Hypothesis 1:** For each tobacco product considered, the prevalence of current nicotine pouch use was significantly higher among adults who currently used that product relative to those who did not. In addition, Table 2 shows that the prevalence of current use of nicotine pouches was highest among adults who currently used smokeless tobacco products (10.55%) [followed by those who used heated tobacco (9.96%) products]. These results support **Hypothesis 2:** Among the tobacco products considered, current use of smokeless tobacco is associated with the highest prevalence of current use of nicotine pouches.

**Table 2:** Significant differences in current nicotine pouch use between adults who use vs. do not use another tobacco product (n = 107,281; N = 241,807,760). Analysis of 2022−23 Tobacco Use Supplement to the Current Population Survey data.

Tobacco Product	Current Use of the Tobacco Product^a^	
Yes % (SE, %)	**No** **% (SE, %)**
Cigars: regular cigars, cigarillos, and little filtered cigars	**3.38 (0.49)**	**0.40 (0.02)**
Combustible (regular) cigarettes	**1.17 (0.12)**	**0.41 (0.03)**
E-cigarettes: vape-pens, e-hookahs, vapes, and modes	**3.16 (0.51)**	**0.39 (0.02)**
Heated tobacco: tobacco vapes with capsules or sticks	**9.96 (3.49)**	**0.46 (0.03)**
Hookah tobacco: water (hookah) pipes filled with tobacco	**4.43 (1.52)**	**0.46 (0.03)**
Regular pipes filled with tobacco	**2.77 (1.25)**	**0.47 (0.03)**
Smokeless tobacco: moist snuff, dip, spit, and chew tobacco, and snus	**10.55 (1.11)**	**0.35 (0.02)**
**At least one of these tobacco products**	**2.06 (0.17)**	**0.22 (0.02)**

^a^ All values are given in percent (%); “SE” stands for standard error; Significant differences are bold (all p-values<0.001).

Table 3 presents the results from the model-assisted analysis. Model fit statistics and additional details are provided in the table footnote. All measures of current use of other tobacco products, except for current use of regular tobacco pipes, were significantly associated with current use of nicotine pouches. Therefore, the model results partially support **Hypothesis 1**. In addition, the highest odds ratio was observed for current use of smokeless tobacco, supporting **Hypothesis 2.**

**Table 3:** Results based on the design-specific multiple logistic regression model (n = 107,281; N = 241,807,760). Analysis of 2022−23 Tobacco Use Supplement to the Current Population Survey data.

Predictor^a^	Odds Ratio	95% Simultaneous Confidence Band^b^
**Current use of**		
Cigars **(p<0.001)**: yes vs. no	**2.44**	**1.70-3.51**
Combustible cigarettes **(p<0.001)**: yes vs. no	**1.87**	**1.41-2.47**
E-cigarettes **(p<0.001)**: yes vs. no	**2.89**	**1.82-4.60**
Heated tobacco **(p=0.001)**: yes vs. no	**4.68**	**1.95-11.25**
Hookah tobacco **(p=0.003)**: yes vs. no	**3.9**	**1.55-8.75**
Regular pipes filled with tobacco (not significant)	0.57	0.13-2.46
Smokeless tobacco **(p<0.001)**: yes vs. no	**12.04**	**8.83-16.43**
**Age (p<0.001)**		
18-24 vs. 65+	**4.92**	**2.20-10.99**
25-44 vs. 65+	**4.41**	**2.19-8.88**
45-64 vs. 65+	**2.19**	**1.10-4.37**
**Sex (p<0.001):** male vs. female	**5.46**	**3.81-7.81**
**Race/ethnicity (p<0.001)**		
Hispanic vs. NH White	**0.31**	**0.16-0.60**
NH Black/African American vs. NH White	**0.35**	**0.16-0.78**
NH Other vs. NH White	**0.17**	**0.08-0.41**
**Highest Level of Education (p=0.038)**		
Less than high school vs. graduate degree or equivalent	1.30	0.59-2.88
High school or equivalent vs. graduate degree or equivalent	1.60	0.90-2.86
Some college or bachelor’s degree vs. graduate degree or equivalent	**1.86**	**1.10-3.15**
**U.S. Region of Residence (p<0.001)**		
Midwest vs. Northeast	1.13	0.72-1.78
South vs. Northeast	0.82	0.52-1.29
West vs. Northeast	**1.73**	**1.08-2.79**

^a^ Survey mode had 331 missing values (see Table 1) and was not significantly associated with current nicotine pouch use (Rao-Scott test p = 0.1486), thus, it was not included in the model to preserve the full sample size; The “NH Other” racial/ethnic category included NH American Indian/Alaska Native, NH Asian/Asian American, NH Hawaiian/Pacific Islander, and NH Multiracial groups due to small sample sizes for some cross-groups: only 1 NH American Indian/Alaska Native adult, 2 NH Asian/Asian American adults, and 1 NH Hawaiian/Pacific Islander adult currently used nicotine pouches; Metropolitan area of residence included two categories (metropolitan, non-metropolitan/unknown), because there were only 3 respondents who reported that they were currently using nicotine pouches for whom the area was “unknown”; The model demonstrated adequate fit: Likelihood Ratio F(22.91; 3,666.13)=50.18, p-value<0.001, with 83.1% concordant, 8.8% discordant, and 8.1% tied observations; Among the control variables, marital status (p = 0.106), employment status (p = 0.770), annual family income (p = 0.067), and metropolitan area of residence (p = 0.776) were not statistically significant: detailed results are not shown.

^b^ Statistically significant results are bold

Table 3 also shows that, after adjusting for other characteristics, the odds of current nicotine pouch use differed significantly by age, sex, race/ethnicity, highest level of education, and U.S. region of residence.

### Secondary goals: characteristics of adults who currently use nicotine pouches

The reduced sample (n = 537) of adults who currently used nicotine pouches represented an estimated 1,146,681 adults in the U.S. population. Table 4 illustrates the prevalence of co-use, defined as current use of nicotine pouches and at least one other tobacco product, for adults who currently use nicotine pouches and for the overall adult population. Compared with the overall adult population, in which combustible cigarettes were the most commonly used tobacco product, the pattern of tobacco use among adults who used nicotine pouches appears different.

**Table 4:** Prevalence of use of other tobacco products among adults who currently use nicotine pouches (n = 537; N = 1,146,681) and All Adults (n = 107,281; N = 241,807,760). Analysis of 2022−23 Tobacco Use Supplement to the Current Population Survey data.

Currently Used Tobacco Product	Adults Who Currently Use Nicotine Pouches % (SE, %)	All Adults % (SE, %)
Cigars	17.30 (2.25)	2.43 (0.06)
Combustible cigarettes	21.74 (1.99)	8.81 (0.11)
E-cigarettes	20.54 (2.82)	3.08 (0.09)
Heated tobacco	2.67 (0.98)	0.13 (0.02)
Hookah tobacco	3.67 (1.26)	0.39 (0.03)
Regular pipes filled with tobacco	1.39 (0.63)	0.24 (0.02)
Smokeless tobacco	27.39 (2.42)	1.23 (0.04)
At least one of the above tobacco products	61.00 (2.59)	14.02 (0.16)

In terms of sociodemographic characteristics (see Table 1 for groups considered), adults who currently used nicotine pouches were predominantly: 25–44 years old (52.98%, SE = 3.02%), 45–64 years old (22.32%, SE = 2.12), or 18–24 years old (19.53%, SE = 2.83); male (89.14%, SE = 1.69%); NH White (85.88%, SE = 2.24%), Hispanic (7.27%, SE = 1.58%), or NH Black/African American (4.59%, SE = 1.44). They were most commonly never married (44.88%, SE = 2.82%) or currently married (39.39%, SE = 2.77%); had attended college or held a Bachelor’s degree (55.66%, SE = 3.16%) or completed High School or equivalent (32.93%, SE = 2.89%); were employed (81.54%, SE = 2.06%) or not in the labor force (15.11%, SE = 1.83%); and reported an annual family income of at least $75,000 (58.96%, SE = 3.18%), $40,000–74,999 (20.34%, SE = 2.18%), or $20,000–39,999 (14.48%, SE = 1.77%). Most lived in the West (30.45%, SE = 2.86%), Midwest (27.80%, SE = 2.28%), or South (27.04%, SE = 2.68%); and resided in metropolitan areas (80.10%, SE = 2.50%). These distributions seemed to differ from those reported in the literature for adults who currently use e-cigarettes or hookah tobacco [8]. For example, while the age and race/ethnicity distributions among adults who currently use nicotine pouches were somewhat similar to those observed for adults who used e-cigarettes, the distribution of educational attainment more closely resembled that of adults who currently use hookah tobacco. Moreover, the distribution of sex appeared especially distinctive: men comprised nearly 90% of adults who used nicotine pouches.

## Discussion

### Key findings

The findings suggest that current use of nicotine pouches was more prevalent among adults who used cigars, combustible cigarettes, e-cigarettes, heated tobacco, hookah tobacco, and smokeless tobacco compared to those who did not use the respective product. Among considered tobacco products, smokeless tobacco use was most strongly associated with nicotine pouch use, while combustible cigarette use showed the weakest association (excluding regular tobacco pipes). Our findings that current nicotine pouch use was more common among adults who used smokeless tobacco, e-cigarettes, or combustible cigarettes are consistent with prior literature [6,7]. The observed associations indicate that use of other tobacco products, particularly smokeless and heated tobacco, may be linked to nicotine pouch use.

Our findings on sociodemographic disparities in the odds of current use of nicotine pouches are consistent with prior reports [5,7], and our estimates of the overall prevalence of current use of each tobacco product (see Table 4) align with those from the National Cancer Institute [15].

Our secondary analysis, limited to adults who currently use nicotine pouches, estimated that approximately 1.15 million adults in the U.S. currently use nicotine pouches. In this population, the estimated prevalence of use of at least one other tobacco product (along with nicotine pouches) was 61.00%, with the most commonly used products being smokeless tobacco, combustible cigarettes, e-cigarettes, and cigars. Other products, such as heated tobacco, hookah tobacco, and regular tobacco pipes, were less commonly used (each with a prevalence below 4.00%). Thus, among adults who use nicotine pouches, poly-tobacco use is common, and tobacco use patterns appear to differ from those in the overall adult population, where combustible cigarettes are the most commonly used product. These findings potentially highlight the need to consider poly-tobacco use patterns in future research targeting adults who use nicotine pouches.

In addition, adults who use nicotine pouches appeared to be primarily 25–44 years old, male, non-Hispanic White, employed, and had relatively high levels of education and income. This sociodemographic profile appeared to differ from that of the overall adult population and from the profile of adults who currently use e-cigarettes or hookah tobacco [8]. These patterns suggest that nicotine pouches may be reaching a unique population of adults, highlighting the importance of considering sociodemographic differences when designing interventions for this group.

### Limitations and future research directions

Our study has several limitations. First, although including survey mode as a control variable is generally recommended [13], we did not include it in the model to preserve the full sample size. The prevalence of current nicotine pouch use between phone interviews (0.53%, SE = 0.05%) and in-person interviews (0.44%, SE = 0.03) did not differ significantly (Rao-Scott chi–squared = 2.07, df = 1, p = 0.1486; n = 106,950), suggesting that survey mode is unlikely to confound the model estimates. Second, while we aimed to consider all racial/ethnic groups separately, small sample sizes required combining some groups of color, so these estimates should be interpreted with caution. Third, our analysis of tobacco use and sociodemographic characteristics among adults who currently use nicotine pouches was limited to descriptive statistics, some based on very small sample sizes (e.g., only eight adults who used nicotine pouches also used regular tobacco pipes). Likewise, comparisons of the sociodemographic profile of adults who currently use nicotine pouches with those of adults who currently use e-cigarettes or hookah tobacco should be considered preliminary, as they are based on a prior study using 2014−15 data [8], which may no longer accurately reflect the current sociodemographic characteristics of adults who use e-cigarettes or hookah tobacco. Fifth, while we applied the TUS-CPS weights correctly in all analyses, we did not perform a formal sensitivity analysis to assess potential variability due to weighting assumptions. Because the U.S. Census Bureau recommends using these weights to produce valid population-level estimates, it would not be appropriate to ignore the weights [20]. To address this limitation, we validated our estimates by comparing them with values reported in the published literature, which provides reassurance regarding the robustness of our findings. Finally, the cross-sectional nature of the data limits the study’s inferences, as causial effects, including transitions between tobacco products, cannot be established.

Future studies should examine disparities in nicotine pouch use across diverse racial and ethnic groups, as use may be more common in certain populations. Future studies should also investigate behavioral transitions in tobacco use, such as whether nicotine pouch use contributes to smoking cessation or serves as a gateway to the use of other tobacco products.

## Conclusions

Our findings highlight the potential role of current use of other tobacco products in nicotine pouch use among U.S. adults. Among the tobacco products considered, nicotine pouch use was most prevalent among adults who use smokeless or heated tobacco, suggesting that use of these products may be more strongly associated with nicotine pouch use than use of other tobacco products. These findings may have implications for future research on public health messaging and tobacco-use prevention efforts.

Furthermore, the high prevalence of use of other tobacco products among adults who currently use nicotine pouches may raise concerns that nicotine pouches could sustain or reinforce poly-tobacco use rather than serve as harm-reduction products. More research is needed to determine the role of nicotine pouches in reducing nicotine dependence from smoking.

The observed differences in sociodemographic characteristics between adults who use nicotine pouches and those who use e-cigarettes or hookah tobacco suggest that nicotine pouches may appeal to distinct sociodemographic groups relative to other tobacco products. It is therefore encouraging that U.S. agencies have begun including nicotine pouch use in national surveillance systems, as this will support more accurate monitoring of emerging patterns in tobacco use behaviors. Given the rapid growth in popularity of nicotine pouches and the evolving tobacco product landscape, ongoing research is critical to assess long-term health impacts, patterns of initiation, and transitions between products. Such evidence will help inform regulatory policies and tailored public health strategies to determine whether nicotine pouch use leads to product switching, cessation, or sustained poly-tobacco use.
